# Bottom-Up and Top-Down Dynamics in the Management of Rosy Apple Aphid

**DOI:** 10.3390/insects16111134

**Published:** 2025-11-06

**Authors:** Ammar Alhmedi, Tim Belien, Dany Bylemans

**Affiliations:** 1 Zoology Department, Research Station for Fruit (Pcfruit Npo), Fruittuinweg 1, 3800 Sint-Truiden, Belgium; tim.belien@pcfruit.be (T.B.); dany.bylemans@pcfruit.be (D.B.); 2 Department of Biosystems, KU Leuven, 3001 Leuven, Belgium

**Keywords:** performance, preference, interspecific interaction, *Aphidius matricariae*, *Aphidius ervi*, *Dysaphis plantaginea*, apple cultivars

## Abstract

Effective control of the rosy apple aphid, *Dysaphis plantaginea*, is crucial for maintaining apple orchard productivity. Understanding plant-mediated bottom-up and parasitoid-driven top-down interactions is critical for developing optimized pest management strategies. We investigated how host plant diversity and interactions between two parasitoids, *Aphidius matricariae* and *Aphidius ervi*, shape aphid suppression across seedlings from ten apple cultivars. Mummy counts, parasitism rate, emergence rate, and sex ratio measurements were used to assess the parasitoid preference and performance. Results revealed significant interactions between host plant identity and parasitoid performance. Mixed parasitoid releases outperformed single-species treatments. Alone, *A. ervi* achieved significantly higher parasitism rates than *A. matricariae*. Host plant effects were pronounced; Cripps Pink enhanced *A. matricariae* performance, while Golden Delicious and Red Delicious improved *A. ervi* metrics. Emergence rates and sex ratios varied by plant origins, with Elstar enhancing *A. ervi* and Granny Smith enhancing *A. matricariae*. Choice tests indicated cultivar-specific parasitoid preferences, and heatmap analysis revealed apparent competition among aphids mediated by parasitoids, with Braeburn and Gala acting as key parasitoid sources. Study findings indicate that managing apple cultivar diversity and exploiting complementary parasitoid interactions can improve *D. plantaginea* biocontrol in orchards.

## Simple Summary

Managing rosy apple aphids is essential for healthy apple orchards. In this study, we examined how combining different apple cultivars with two natural enemies, *Aphidius matricariae* and *Aphidius ervi*, affects aphid control. We found that releasing both parasitoid species together provided better control than using either species alone. Additionally, certain apple cultivars improved the effectiveness of one parasitoid species over the other, highlighting that the choice of apple variety can significantly influence pest management outcomes. These findings suggest that farmers can achieve more sustainable and effective aphid control by selecting specific apple cultivars and combining multiple natural enemies, reducing the need for chemical insecticides.

## 1. Introduction

The rosy apple aphid, *Dysaphis plantaginea* Passerini (Hemiptera: Aphididae), persists as a significant pest in apple orchards across Europe and North America, leading to substantial yield losses, particularly in organic production systems, where control relies on a limited range of organically approved insecticides, such as neem-based formulations [1,2,3]. While conventional chemical control remains widely practiced, the increasing drive toward sustainable agriculture has prompted a shift to integrated pest management (IPM) strategies, favoring ecological processes and the minimization of insecticide use. In this context, understanding and leveraging both bottom-up and top-down ecological interactions are essential for optimizing pest management.

Bottom-up trophic effects refer to the influence of primary producers, such as plants, and the resources they provide on higher trophic levels, including herbivores and their natural enemies. In the context of aphid management, these effects encompass plant cultivar identity, nutritional quality, and defensive chemical profiles, all of which regulate aphid population growth, behavior, and susceptibility to natural enemies [4,5,6,7,8,9,10]. In apple systems, cultivars differ significantly in their susceptibility to *D. plantaginea*, directly affecting aphid fecundity, colony establishment, and outbreak potential [3,10,11]. Consequently, cultivar-based variability can shape both *D. plantaginea* abundance and the efficacy of parasitoid- and predator-mediated control, representing a critical factor in sustainable management strategies [12,13,14].

Top-down forces describe the regulatory effects exerted by natural enemies, such as predators and parasitoids, which suppress aphid populations via predation or parasitism. In European orchards, hymenopteran parasitoids such as *Ephedrus persicae* Froggatt, *Aphidius ervi* Haliday, and *Aphidius matricariae* Haliday (Hymenoptera: Braconidae) have been documented as parasitoids of *D. plantaginea* [15,16]. Field studies consistently highlight that *E. persicae* is the dominant parasitoid on *D. plantaginea* populations in apple orchards [1,17]. However, its practical applications in augmentative releases are constrained by an end-spring and summer diapause [18], prompting research into alternative parasitoids that possess traits more suited to sustained production programs and field application. As a result, attention has shifted to other parasitoids, such as *A. ervi* and *A. matricariae*, which exhibit traits better suited to mass rearing and continuous release [19,20]. Laboratory and field studies have demonstrated marked differences in parasitism rate and host stage preference among species, which may determine their suitability across orchard environments [21,22]. Importantly, *A. ervi* and *A. matricariae* often coexist, raising the potential for both interspecific competition and facilitation that may shape their effectiveness in biological control programs [23,24,25].

Recent field and laboratory studies have differentiated the effects of bottom-up and top-down forces under both controlled and natural conditions. These studies reveal that while bottom-up factors such as plant quality and cultivar differences can either promote or inhibit aphid population growth and natural enemy development [7,26], strong top-down pressures exerted from natural enemy assemblages can override bottom-up influences [12,13,14,27]. However, it has been demonstrated that certain secondary plant metabolites can act as repellents against parasitoid attacks or modify aphid behavior, thereby influencing parasitoid foraging success [28,29]. This underscores the necessity of an integrated ecological approach for effective aphid suppression.

Top-down control efficacy is further complicated by interspecific interactions among parasitoid species, especially under resource limitations. Both laboratory and field investigations show that aphid parasitoids engage in direct and indirect competition as well as facilitation, yielding antagonistic or synergistic effects on biological control outcomes. For example, resource competition between species such as the hymenopteran parasitoids *Aphidius colemani* Viereck and *Ephedrus cerasicola* Stary (Hymenoptera: Braconidae) can depress parasitism rates under controlled conditions [30,31], while field observations highlight facilitation, where different parasitoid species exploit distinct aphid stages, thereby enhancing overall parasitism [32,33]. Furthermore, co-occurring parasitoid species can improve foraging efficiency by responding collectively to volatile cues emitted by infested plants. However, variability in chemical profiles among different cultivars (bottom-up factors) can sometimes hinder parasitoid performance, as certain cultivars emit volatile organic compounds that may be less attractive to natural enemies [34,35]. For instance, cultivars with lower emissions of attractant compounds may impede parasitoids’ ability to locate aphid-infested plants [36,37].

The concept of apparent competition illustrates the integration of bottom-up and top-down forces in structuring aphid communities and influencing biological control efficacy. Field evidence shows that aphids thriving on favorable cultivars (bottom-up effect) support larger number of natural enemies that can intensify predation or parasitism on aphid populations feeding on less suitable cultivars (top-down effect), even without direct resource competition [38,39,40,41,42]. Controlled experiments confirm that these effects occur both among different aphid species and among populations of the same species on different host plants sharing parasitoids [43,44]. Both field and laboratory studies demonstrate that shared natural enemies mediate such interactions, leading to phenomena like the spillover effect, where natural enemies sustained by abundant aphid populations attack less abundant ones [17,45]. Host preference, demonstrated in choice bioassays, is a pivotal factor driving parasitoid-mediated apparent competition, impacting aphid community structure and biological control outcomes [8,46]. Thus, apparent competition presents both opportunities and challenges for aphid pest management [47,48,49]. Although the ecological complexity complicates precise predictions, integrated management approaches that explicitly consider and combine bottom-up and top-down forces are essential [50,51]. Addressing the multitrophic interactions among host plants, aphids, and natural enemies forms a fundamental basis for sustainable and enhanced biological control strategies against *D. plantaginea* in apple orchards.

In this study, we investigated how host plant variation (bottom-up effects) and interspecific interactions between parasitoids (top-down effects) influence the performance and preference of the parasitoids *A. matricariae* and *A. ervi* against the rosy apple aphid *D. plantaginea*. Specifically, our objectives were to: (1) determine how genetically variable host plants infested with aphids influence parasitoid performance (bottom-up effect); (2) assess how interspecific interactions between *A. matricariae* and *A. ervi* affect their efficiency against aphids (top-down effect); (3) evaluate whether parasitoids exhibit host-plant related preferences when provided with aphid-infested seedlings of different genetic origin; and (4) examine the extent to which the apparent competition occurs between aphid populations on different host plants and how this shapes parasitoid foraging outcomes (integration of bottom-up and top-down dynamics). To address these objectives, we used apple seedlings grown from seeds originating from ten apple cultivars. While the seedlings are not genetically identical to the cultivars of origin, previous studies have demonstrated that variation at the cultivar level affects rosy apple aphid fitness [3,11]. By using genotypically variable seedlings, we aimed to capture the influence of host plant genetic differences on aphid–parasitoid interactions. Parasitoid performance was quantified using key metrics including mummy counts, parasitism rate, emergence rate, and sex ratio. Parasitoid preference was estimated through mummy counts in multi-host plant choice experiments. We also conducted choice tests with aphid-infested seedlings of different genotypes to assess how plant–aphid associations mediate parasitoid foraging and potential apparent competition.

## 2. Materials and Methods

### 2.1. Apple Seedlings

Fruits were collected from ten apple, *Malus domestica* L., cultivars—Braeburn, Cox, Cripps Pink, Elstar, Gala, Golden Delicious, Granny Smith, Kanzi, Red Delicious, and Topaz—at the Fruit Research Center (pcfruit) in Sint-Truiden, Limburg, Belgium. Seeds extracted from these fruits were germinated, and the seedlings, approximately 10 cm in height, were individually potted in containers measuring 8.5 cm in diameter and 9 cm in height, using commercial potting soil. For clarity and brevity, seedlings originating from the seeds of each cultivar’s fruits are hereafter referred to by the cultivar name. It should be noted, however, that these seedlings are not genetically identical to the original cultivars due to genetic recombination. The plants were maintained in a controlled greenhouse environment with a temperature of 22 ± 2 °C, a 16:8 h light–dark photoperiod, and 60 ± 5% relative humidity. To support growth, NPK 10-52-10 was applied during the first two weeks to promote root development, followed by NPK 20-20-20 to enhance foliar growth. Following transplantation, seedlings received a single application of NPK fertilizer (10-52-10) during the first two weeks to promote root development. To prevent infection by powdery mildew, the fungicide Luna Privilege (fluopyram; Bayer CropScience, Monheim am Rhein, Germany) was applied two weeks after transplantation at the manufacturer’s recommended concentration of 0.6 mL per liter of water. Thereafter, NPK fertilizer (20-20-20) was applied in the third and fourth weeks to stimulate vegetative growth. Irrigation was performed at intervals of 2–3 days.

### 2.2. Aphids

A colony of *D. plantaginea* was established from a field population collected in 2020 from a Topaz-cultivar apple orchard at the Fruit Research Center (pcfruit) in Sint-Truiden, Limburg, Belgium. The aphids were maintained under controlled laboratory conditions (22 ± 2 °C, 60 ± 5% relative humidity, 16:8 h light–dark photoperiod) on Boskoop-cultivar apple seedlings for at least two months prior to experimentation. Groups of more than 100 reproductive adults were transferred onto healthy one-year-old Boskoop apple trees to feed and reproduce. Nymphs produced within 96 h of introduction were collected and used in subsequent experiments.

### 2.3. Parasitoids

Two commercially available parasitoid species, *A. ervi* and *A. matricariae*, were used in this study. The parasitoid specimens were supplied in the form of mummies by Biobest (Westerlo, Belgium). Upon receipt, the mummies were transferred to 94 mm diameter Petri dishes and placed within mesh cages, which were maintained under environmental conditions identical to those used for aphid rearing. To sustain the emerging adult parasitoids, a 2% sugar water solution was provided. Mummies were checked daily for adult emergence. Two days prior to trials, cohorts of newly emerged parasitoids were grouped in a 1:2 female-to-male ratio in aerated tubes (3 cm diameter × 6.5 cm height) to ensure mating. A cotton ball soaked in a 2% sugar solution was placed inside the tubes to serve as food for the adult parasitoids.

### 2.4. Experimental Design

All experiments were conducted in a research greenhouse under controlled conditions (22 ± 2 °C, 60 ± 5% RH, 16:8 h light–dark photoperiod) using ventilated cages. The front and back panels were constructed of transparent plastic to allow observation of insect activity and light penetration, while the remaining sides were made of polyester mesh netting to ensure ventilation. Each experiment consisted of ten replicates. Four experimental scenarios were tested: (i) single parasitoid species with single aphid-infested seedling genotype, (ii) single parasitoid species with multiple aphid-infested seedling genotypes, (iii) mixed parasitoid species (*A. ervi* and *A. matricariae*) with single aphid-infested seedling genotype, (iv) mixed parasitoid species with multiple aphid-infested seedling genotypes. One day prior to trials, 50 mixed-instar *D. plantaginea* nymphs were introduced to each seedling (20–25 cm height) using an infested leaf piece technique [10]. This method was employed to minimize damage to aphid stylets and facilitate natural colonization of the test plants. Aphid counts were standardized to 50 per plant 1–2 h before parasitoid exposure. Mummies and live aphids found on apple seedlings were counted two weeks after parasitoid release. The mummies were then transferred to a climate-controlled chamber and incubated for one week at 22 ± 1 °C, 16:8 h light–dark photoperiod and 60% ± 5% relative humidity until adult emergence.

Each experiment consisted of ten replicates per treatment combination, a number chosen to balance statistical power with logistical feasibility. Previous laboratory studies with aphid-parasitoid systems have demonstrated that around ten replicates typically provide sufficient replication to detect treatment effects while minimizing noise from biological variability [46,52]. This level of replication also ensured adequate degrees of freedom for the factorial structure of our experimental scenarios, increasing the robustness of comparisons across host plant genotypes and parasitoid assemblages [52,53].

### 2.5. Experiment 1: Single Parasitoid Species with Mono-Cultivar System

This experiment evaluated the impact of various aphid-infested apple seedlings on mummy count, parasitism rate, emergence rates, and sex ratios of *A. matricariae* and *A. ervi*. Two mated females of each parasitoid species were released into separate cages (24.5 × 24.5 × 63 cm) containing a single aphid-infested potted apple seedling. The mummies and aphids were counted after 14 days, followed by an assessment of the parasitoid adult emergence rate and sex ratio after a previously described incubation period.

### 2.6. Experiment 2: Single Parasitoid Species with Multi-Cultivar System

We evaluated the host preference of *A. ervi* and *A. matricariae* for parasitizing aphids on various apple seedlings within a multi-cultivar environment. For each parasitoid species, two mated females were released into a cage (47.5 × 47.5 × 93 cm) containing ten potted apple seedlings, each representing a different cultivar and infested with aphids. Fourteen days after the release, the number of mummified aphids on each apple cultivar was recorded.

### 2.7. Experiment 3: Mixed Parasitoid Species with Mono-Cultivar System

The impact of interspecific interactions on parasitoid performance was investigate. One mated female of each parasitoid species was released into a cage (24.5 × 24.5 × 63 cm) containing a single aphid-infested potted apple seedling. Mummy count, parasitism rate, and emerged adult numbers were assessed after 14 days. Performance metrics were compared between *A. ervi* and *A. matricariae* across apple cultivars and overall. Results were also compared to the single parasitoid species condition from Experiment 1. Adult parasitoids that emerged in mixed-species treatments were identified to species using morphological keys [54,55] under the stereomicroscope.

### 2.8. Experiment 4: Mixed Parasitoid Species with Multi-Cultivar System

This experiment assessed the indirect interactions between various aphid-infested seedlings. Two mated females of each parasitoid species were released into cages (47.5 × 47.5 × 93 cm) containing ten aphid-infested potted apple seedlings, each representing a different cultivar. The mummies and aphids were counted after 14 days after parasitoid release. The emerged parasitoid adults were counted per species for each plant-aphid combination, followed by an assessment of the parasitoid adult emergence rate after a previously described incubation period. The identification of adult parasitoids was carried out using the morphological keys mentioned above. Potential apparent competition was evaluated based on mummy and aphid counts per cultivar.

### 2.9. Data Analysis

All statistical analyses were performed following a stepwise approach corresponding to the response variables. Prior to analysis, all datasets were tested for normality and variance homogeneity using Minitab 18 software [56]. Non-normal data were log10-transformed to meet parametric assumptions; those still not meeting normality assumptions were analyzed with non-parametric tests. Differences in mummy production across treatments and cultivars was analyzed using generalized linear models (GLM), followed by Tukey’s HSD post hoc comparisons (*p* ≤ 0.05). In addition, a Principal Component Analysis (PCA) was performed in XLSTAT 2019 software [57] to visualize relationships among apple cultivars and parasitoid treatments based on the number of mummies produced by *A. ervi* and *A. matricariae*. Parasitism rate was calculated as the number of mummies divided by the total number of mummies and living individuals. To evaluate cultivar-driven effects, datasets were analyzed using the non-parametric Kruskal–Wallis test followed by Dunn’s pairwise comparisons (*p* ≤ 0.05), whereas interspecific interaction effects were assessed using GLM followed by Tukey’s HSD test (*p* ≤ 0.05). PCA was also conducted to explore distribution variability of parasitism success across cultivar–aphid–parasitoid associations, with the first axes retained to explain the majority of variance.

Emergence rate was computed as the proportion of emerged adults relative to the total number of mummies. Differences among cultivars were analyzed using the Kruskal–Wallis test (*p* ≤ 0.05), and Dunn’s test was employed for post hoc multiple comparisons. Sex ratio data (% males) were analyzed using Kruskal–Wallis (*p* ≤ 0.05), followed by Dunn’s test for multiple comparisons across cultivars in order to detect variation in progeny sex allocation.

To investigate parasitoid preference across host plant–aphid associations, GLM with Tukey’s HSD (*p* ≤ 0.05) was applied to the number of mummies produced. Additionally, a hierarchical clustering analysis was performed in XLSTAT 2019 software to construct a dissimilarity dendrogram of parasitoid responses. Pairwise dissimilarities were based on Euclidean distances, and clustering was achieved using the unweighted pair-group method with arithmetic mean (UPGMA). This approach enabled the identification of distinct response clusters for *A. ervi* and *A. matricariae* across aphid-infested apple cultivars.

Parasitoid-mediated indirect interactions between host plant-aphid associations were visualized using R Studio software version 4.4.1 [58]. A heatmap with a color gradient was employed to represent the strength of parasitoid overlap between different cultivar-aphid associations. The strength of indirect interactions between cultivar-aphid associations via parasitoids was assessed using the formula described by Müller et al. [45]. All computing of indirect interaction indices were performed in Mathematica 5.0 [59]. The color gradient of the heatmap is indicative of the quantitative index of potential parasitoid sharing, ranging from 0 (no potential) to 1 (maximum potential). A dendrogram tree was also constructed and combined with the heatmap to illustrate the dissimilarity between aphid-infested cultivars in terms of their potential as sources of parasitoids attacking other aphid-infested cultivars. This mathematical approach helps quantify how parasitoids connect aphid populations across various apple cultivars and elucidates how multi-cultivar systems influence parasitoid-mediated aphid suppression.

## 3. Results

### 3.1. Mummy Production

#### 3.1.1. Cultivar-Driven Bottom-Up Effects

For both parasitoid species, significant variation in parasitoid success, measured by mummy formation, was observed across apple cultivars infested with rosy apple aphids (GLM: *A. ervi*, F_9,90_ = 11.32, *p* < 0.001; *A. matricariae*, F_9,90_ = 9.8, *p* < 0.001; Table 1). For *A. ervi*, the highest mean number of mummies occurred on Golden Delicious (24.4) and Red Delicious (18.2), which significantly (Tukey’s HSD, *p* ≤ 0.05) outperformed cultivars such as Gala (6.4) and Kanzi (5.2). Intermediate parasitism success occurred on Braeburn (13.7), Cox (13.2), and Topaz (13.0), which did not statistically differ from the top-supporting cultivars. *A. matricariae* exhibited divergent success patterns, peaking on Cripps Pink (17.9 mummies). Braeburn (10.3) and Topaz (8.1) showed moderate success, while Golden Delicious (4.1) and Gala (4.3) were least favorable. This inverse relationship between the two parasitoid species highlights host plant-mediated trade-offs in parasitism outcomes.

#### 3.1.2. Interspecific Interaction Effects

**Figure 1:**
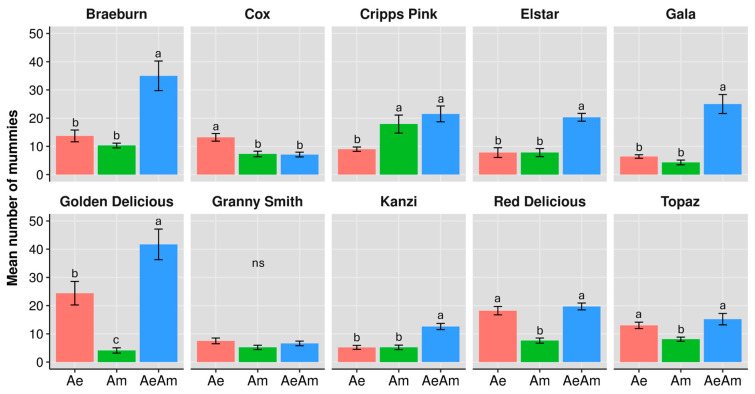
Number of mummies (mean ± SE) of study parasitoid species, when they are alone *Aphidius ervi* (Ae, red bars), *Aphidius matricariae* (Am, green bars), and when they are co-released (AeAm, blue bars), on rosy apple aphid *Dysaphis plantaginea* infesting different apple cultivars in mono-cultivar system. Different letters indicate significant differences between parasitoid presence scenarios (GLM and Tukey test, *p* ≤ 0.05).

Statistical analysis using Generalized Linear Model and Tukey post hoc test revealed differences amongst parasitoid treatments in mummy formation across most aphid-infested apple cultivars (Figure 1). When *A. ervi* and *A. matricariae* were co-released, their combined performance often exceeded their solitary performances, particularly on Braeburn and Golden Delicious. For the cultivar Braeburn, the combined presence of *A. ervi* and *A. matricariae* resulted in higher counts of mummies (35.0), compared to their solitary performances (13.7 for *A. ervi* and 10.3 for *A. matricariae*, F_2,27_ = 20.32, *p* < 0.001). Golden Delicious exhibited the highest impact of interspecific interaction (F_2,27_ = 60.09, *p* < 0.001) with a combined mummy count of 41.7 compared to solitary performances of *A. ervi* (24.4) and *A. matricariae* (4.1). In contrast, on Cox cultivar, interspecific interaction did not enhance mummy formation. The combined treatment produced fewer mummies (7.1) compared to *A. ervi* alone (13.2), indicating potential competitive interference or resource limitation under joint parasitism conditions (F_2,27_ = 7.84, *p* = 0.002). Notably, *A. matricariae* outperformed *A. ervi* on Cripps Pink (17.9 vs. 9.0; F_2,27_ = 10.56, *p* < 0.001), though the combined treatment (21.5) showed no significant advantage over *A. matricariae* alone. Conversely, *A. ervi* dominated on Red Delicious (18.2 vs. 7.6 for *A. matricariae*; F_2,27_ = 30.71, *p* < 0.001), with the combined treatment (19.7) matching the solitary performance of *A. ervi*. For the cultivar Granny Smith, there was no significant difference (F_2,27_ = 1.86, *p* = 0.176) in the number of mummies between the combined presence of both parasitoids (6.6) and when they were alone (7.5 for *A. ervi*, 5.2 for *A. matricariae*).

**Figure 2:**
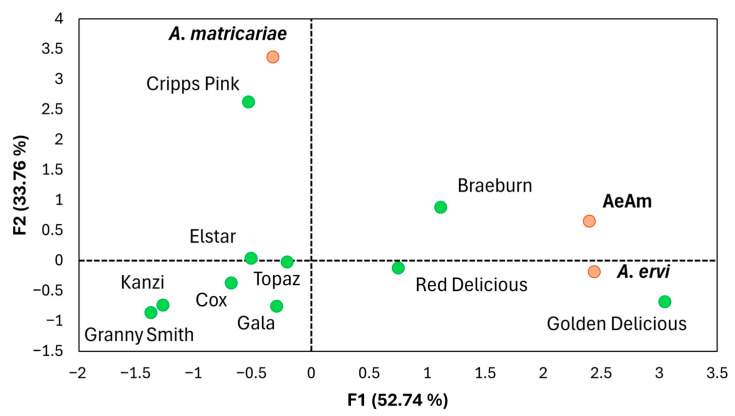
Principal component analysis (PCA) biplot illustrating the relationships among apple cultivars and parasitoid treatments based on the number of mummies of *Aphidius ervi* and *Aphidius matricariae* on rosy apple aphid. AeAm = *A. matricariae* and *A. ervi* together in mono-cultivar system. The apple cultivars are represented by green points, while the parasitoids are marked in orange. Principal components F1 (52.74%) and F2 (33.76%) are displayed.

The principal component analysis (PCA) biplot demonstrated distinct clustering of aphid-infested cultivars relative to the parasitoids. The two principal components, F1 and F2, explain 52.74% and 33.76% of the total variance, respectively, providing insights into the relationships between apple cultivars and parasitoid performance (Figure 2). Cultivars such as Granny Smith, Kanzi, Cox, Gala, Topaz, and Elstar are positioned in the negative F1 region, indicating lower numbers of mummies associated with these cultivars compared to others. In contrast, cultivars like Braeburn, Red Delicious, and Golden Delicious are located in the positive F1 region closer to mixed parasitoid species and *A. ervi*, suggesting higher mummy formation and a stronger association with these parasitoids. Additionally, Cripps Pink is positioned in the positive F2 region near *A. matricariae*, highlighting a specific relationship with this parasitoid species.

### 3.2. Parasitism Rate

#### 3.2.1. Cultivar-Driven Bottom-Up Effects

Significant bottom-up effects of apple cultivars on parasitism rates were observed for both *A. ervi* (KWT: H = 64.95, df = 9, *p* < 0.001) and *A. matricariae* (KWT: H = 52.03, df = 9, *p* < 0.001). For *A. ervi*, the highest parasitism rates were observed on Golden Delicious (38.7%) and Red Delicious (31.1%), which were statistically different compared to most other cultivars in this study (Table 2). An intermediate parasitism rate (23.0%) of this parasitoid was observed on Braeburn, which did not differ from Red Delicious or Golden Delicious. In contrast, the lowest parasitism rates for *A. ervi* were recorded on Gala (8.0%), Granny Smith (7.8%), and equally on Cripps Pink and Kanzi (both 6.4%), all belonging to the lowest statistical groups (cd or d, Table 2).

Similarly to *A. ervi*, the parasitism rate of *A. matricariae* was influenced by the apple cultivar (Table 2). However, the pattern of cultivar effects differed between the two parasitoid species, especially for Cripps Pink. The highest parasitism rate for *A. matricariae* was recorded on Cripps Pink (24.3%), which was significantly higher than all other cultivars except Cox (14.3%). The lowest parasitism rate for *A. matricariae* was observed on ‘Gala’ (4.7%), which was statistically distinct from most other cultivars (Dunn’s test, Table 2). The cultivars Elstar, Red Delicious, and Braeburn supported moderate parasitism rates (11.4%, 11.2%, and 9.7%, respectively).

#### 3.2.2. Interspecific Interaction Effects

**Figure 3:**
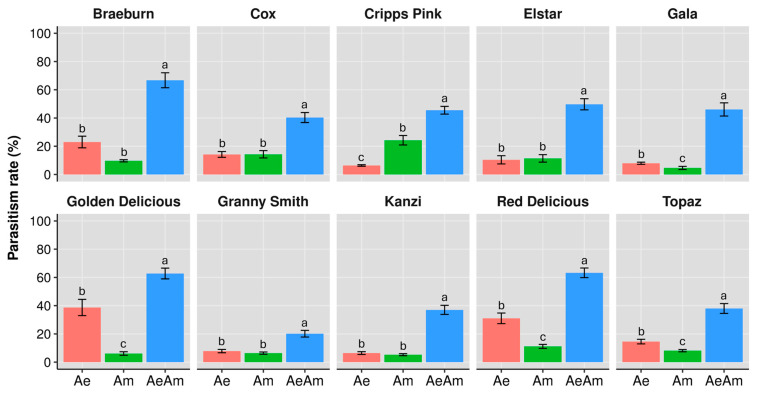
Parasitism rate (% ± SE) of study parasitoid species, when they are alone *Aphidius ervi* (Ae, red bars), *Aphidius matricariae* (Am, green bars), and when they are co-released (AeAm, blue bars) on rosy apple aphids across different apple cultivars in mono-cultivar system. Different letters indicate significant differences between parasitoid presence scenarios (GLM and Tukey test, *p* ≤ 0.05).

The parasitism rates of *A. ervi* and *A. matricariae* under solitary and co-released conditions exhibited significant variations across apple cultivars (GLM, all *p* < 0.001). Co-release of both parasitoid species consistently resulted in synergistic effects, with parasitism rates surpassing solitary treatments across all study cultivars (Figure 3). On Braeburn, *A. ervi* achieved 23.0% parasitism, (F_2,27_ = 63.15, *p* < 0.001) lower than the co-released condition (66.7%), while *A. matricariae* alone showed minimal efficacy (9.7%). Similar patterns emerged in Golden Delicious and Red Delicious (Figure 3). Notably, Cripps Pink demonstrated reversed solitary performance, with *A. matricariae* achieving higher parasitism rates (24.3%) than *A. ervi* (6.4%), though co-release still produced the highest efficacy (45.5%; F_2,27_ = 108.32, *p* < 0.001). The lowest co-release parasitism rate was observed on Granny Smith (20.2%), though still higher than solitary conditions (*A. ervi*: 7.8%; *A. matricariae*: 6.4%). Tukey’s post hoc test confirmed significant differences between the co-release condition and solitary releases for both parasitoids across all tested cultivars (Figure 3).

**Figure 4:**
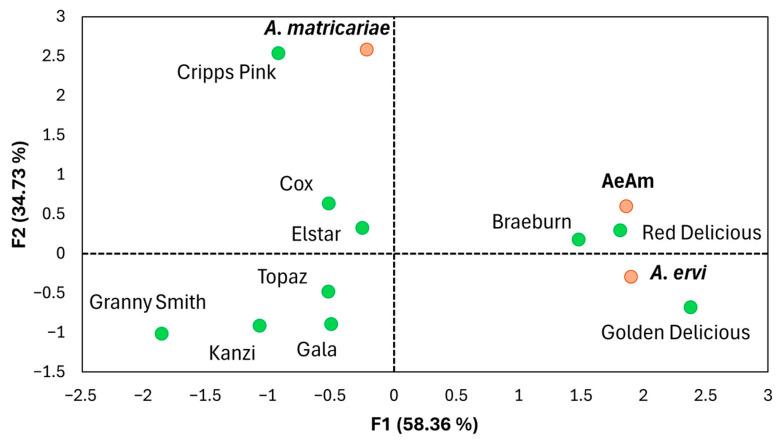
Principal component analysis (PCA) biplot illustrating the relationships among apple cultivars and parasitoid treatments based on the parasitism rates of *Aphidius matricariae* and *Aphidius ervi* on rosy apple aphid. AeAm = *A. matricariae* and *A. ervi* together in mono-cultivar system. The apple cultivars are represented by green points, while the parasitoids are marked in orange. Principal components F1 (58.36%) and F2 (34.73%) are displayed.

Principal component analysis (PCA) further supported the observed variation pattern in parasitism rates across different parasitoid presence scenarios and associated aphid-infested apple cultivars (Figure 4). The first two principal components (F1 = PC1 and F2 = PC2) accounted for 58.36% and 34.73% of the total variance, respectively. Positioned on the positive region of PC2, the cultivars Cripps Pink, Cox and Elstar were separated from the other cultivars and were more associated with *A. matricariae*. The co-release treatment and *A. ervi* alone were positioned on the positive region of PC1, near Red Delicious, Braeburn and Golden Delicious, association with similar patterns of parasitism rates.

### 3.3. Emergence Rate

**Figure 5:**
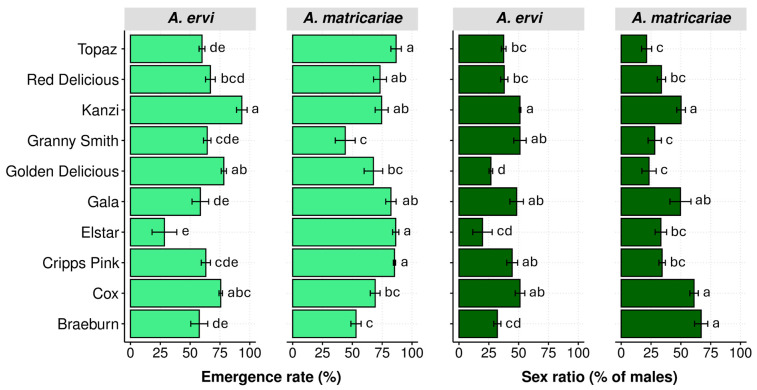
The bottom-up effects of apple cultivars on adult emergence (%, mean ± SE) rates and sex ratios (% males, mean ± SE) of *Aphidius ervi* and *Aphidius matricariae*. Different letters indicate significant differences in the parasitism rates between associated cultivars for each parasitoid species (Kruskal–Wallis and Dunn’s tests, *p* ≤ 0.05).

The adult emergence rates of the two parasitoid species were evaluated across ten apple cultivars in a mono-cultivar system, revealing significant variability influenced by host plant identity (Figure 5). The adult emergence rates of both parasitoid species exhibited significant variation across the studied apple cultivars (Kruskal–Wallis test: *A. matricariae*, H = 44.36, df = 9, *p* < 0.001; *A. ervi*, H = 48.08, df = 9, *p* < 0.001). For *A. matricariae*, adult emergence rates ranged from 44% to 87%. The highest emergence rates were observed on Topaz (87%), Elstar (86%), and Cripps Pink (85%), while the lowest were recorded on Granny Smith (44%) and Braeburn (53%) cultivars. *A. ervi* exhibited a different pattern, with emergence rates varying from 28% to 93%. The most favorable cultivar for *A. ervi* was Kanzi (93%), whereas Elstar (28%) and Braeburn (58%) yielded the lowest emergence rates.

### 3.4. Sex Ratio

The data reveal distinct patterns in sex ratio, measured by the percentage of males, between the two parasitoid species across the apple cultivars (Figure 5). Notably, *A. matricariae* showed a wider range of sex ratios (21.3–67.0%) compared to *A. ervi* (19.7–51.1%). The sex ratio of *A. ervi* varied significantly across the study apple cultivars (Kruskal–Wallis test: H = 37.29, df = 9, *p* < 0.001). The highest male percentages were observed on Granny Smith, Cox, and Kanzi (all 51.1%), while the lowest male percentages were on Elstar (19.7%), Golden Delicious (26.7%), and Braeburn (32.1%). Similarly, *A. matricariae* exhibited variation in sex ratio across the apple cultivars (Kruskal–Wallis test: H = 51.98, df = 9, *p* < 0.001). The highest percentages of males were observed on the Braeburn cultivar (67.0%), followed by Cox (61.1%). In contrast, the lowest percentages were recorded on Topaz (21.3%), Golden Delicious (23.3%), and Granny Smith (28.1%).

### 3.5. Parasitoid Preference

**Figure 6:**
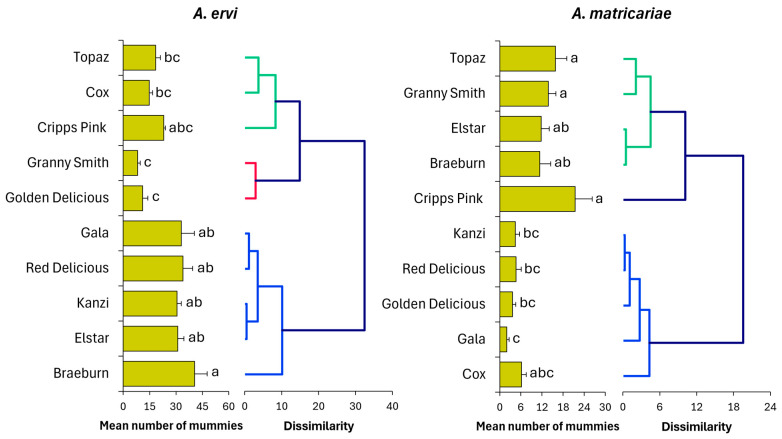
Mean number of mummies (±SE) of *Aphidius matricariae* and *Aphidius ervi* on rosy apple aphid *Dysaphis plantaginea* infesting different apple cultivars. Different letters indicate significant differences between parasitoid presence scenarios (GLM and Tukey test, *p* ≤ 0.05). The associated dendrogram visualizing the distinctive clusters of both study parasitoid species was constructed using the number of mummies produced on the different aphid-infested apple cultivars. The dissimilarity pattern established for the analysis was based on the variation in number of mummies among apple cultivars.

Generalized linear model (GLM) indicated significant differences in parasitization preferences of *A. ervi* and *A. matricariae* among *D. plantaginea*-infested apple cultivars (*A. ervi*: F_9,90_ = 10.86, *p* < 0.001; *A. matricariae*: F_9,90_ = 7.68, *p* < 0.001, Figure 6). Post hoc Tukey test (*p* ≤ 0.05) revealed distinct cultivar-specific trends for each parasitoid species. For *A. ervi*, Braeburn followed by Red Delicious and Gala exhibited the highest numbers of mummified aphids (40.6, 34.0, and 33.0, respectively), while cultivars such as Golden Delicious (11.0) and Granny Smith (8.2) showed lower numbers of mummies. Intermediate numbers of mummified aphids were found on other cultivars such as Cox (14.8), Cripps Pink (23.0), and Topaz (18.4 ± 2.7). The parasitoid *A. matricariae* exhibited a different preference pattern. The highest number of mummies per aphid-infested cultivar seedling was observed on Cripps Pink (21.4) followed by Topaz (15.8) and Granny Smith (13.8) cultivars. In contrast, the lowest numbers of mummified aphids were recorded on Gala (2.0), Golden Delicious (3.6), Kanzi (4.4), and Red Delicious (4.6) cultivars.

### 3.6. Parasitoid-Mediated Indirect Interactions

**Figure 7:**
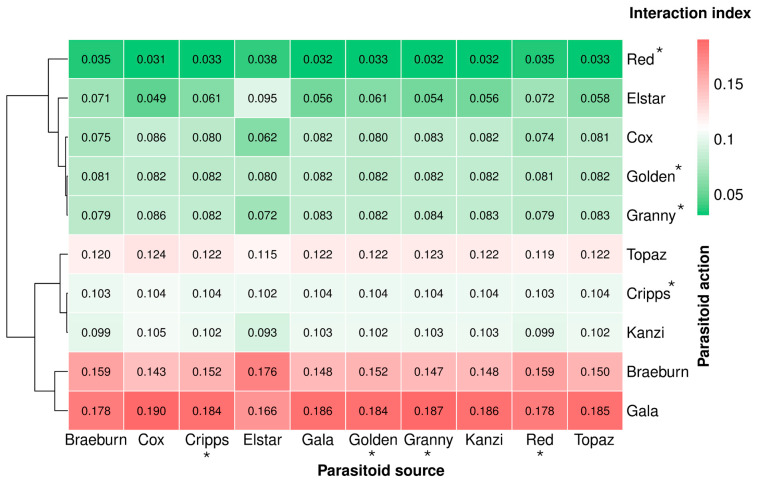
Heatmap illustrating parasitoid-mediated indirect interactions among aphid populations on different apple cultivars. Interaction strengths are represented using a quantitative index of potential parasitoid sharing between aphids infesting various cultivars, with values ranging from 0 (no potential) to 1 (maximum potential). Cultivars with asterisks are: Cripps Pink, Golden Delicious, Granny Smith, and Red Delicious, respectively.

The strength of indirect interactions was visualized using a heatmap with a color gradient corresponding to the quantitative index of potential parasitoid sharing. This index ranged from 0 (no potential) to 1 (maximum potential). The heatmap illustrates the variability in parasitoid-based indirect interaction indices, representing the likelihood of parasitoids attacking aphids on one cultivar while having developed on aphids infesting another cultivar or co-inhabiting aphids on the same host cultivar (Figure 7). The calculated indices, visualized by the heatmap color gradient, revealed varying degrees of parasitoid sharing among aphid-infested apple cultivars, highlighting distinct indirect interactions mediated by shared parasitoid populations. Analysis of these indices demonstrated that parasitoids exhibited both cultivar-specific development patterns and cross-cultivar movement. Among cross-cultivar interactions, the highest level of parasitoid sharing was observed between Gala aphids as a potential source for parasitoids attacking Cox aphids (index: 0.1900), followed by Gala as source for Granny Smith (index: 0.1870). In contrast, the lowest parasitoid sharing was observed when Red Delicious aphids were considered as the source of parasitoids that potentially attacked Cox aphids (0.0310). Overall, Gala aphids exhibited the highest potential as a parasitoid source (average index: 0.1824), followed by Braeburn (0.1534) and Topaz (0.1211). Conversely, Red Delicious (0.0334), Elstar (0.0634) and Cox (0.0787) displayed the lowest source potential, indicating minimal contribution to parasitoid populations affecting other cultivars.

The interaction strengths between aphid-infested cultivars were notably asymmetrical. For example, most parasitoids attacking aphids on Cox have potentially developed on Gala (0.1900), whereas the reciprocal effect of parasitoids attacking aphids on Gala developing on Cox was considerably lower (0.0824). Similarly, parasitoids from Braeburn significantly impacted Elstar (0.1760), but the reverse interaction was weaker (0.0714).

The proportion of parasitoids attacking aphids on their natal cultivar (self-sourcing) ranged from 0.0862 (Cox) to 0.1862 (Gala), with intermediate values observed for Braeburn (0.1591), Cripps Pink (0.1038), Kanzi (0.1030), and Topaz (0.1220). Notably, Gala displayed the highest level of self-sourced parasitoids, suggesting that important number of parasitoids developing on Gala-infesting aphids tended to remain on the same cultivar. In contrast, Red Delicious had the lowest self-sourcing index, indicating substantial parasitoid exchange with other cultivars.

## 4. Discussion

Understanding the complex interplay between host plant characteristics and natural enemy dynamics is fundamental for developing sustainable pest management strategies in agroecosystems. This study elucidates the intricate tritrophic dynamics between apple seedlings, derived from different cultivars, the rosy apple aphid *D. plantaginea*, and its parasitoids *A. ervi* and *A. matricariae*, thereby revealing critical implications for optimizing biological control in apple orchards. While intraspecific variation among apple cultivars is well established as a driver of herbivore performance [3,11,60], its cascading influence on parasitoid effectiveness and reproductive success remains insufficiently explored. Our findings demonstrate that aphid-parasitoid interactions were substantially shaped by the intraspecific variability among host apple seedlings, consistent with the growing body of evidence that plant genotype and quality cascade upward in tritrophic systems [10,61]. These results highlight critical bottom-up and top-down forces shaping biological control outcomes, reinforcing the need to integrate both dimensions in IPM frameworks designed for apple orchards.

The observed differences in parasitoid success across seedling origins underscore the pivotal role of host plant quality in modulating tritrophic interactions [62,63]. Specifically, *A. ervi* consistently outperformed on Golden Delicious and Red Delicious seedlings, whereas *A. matricariae* was more successful on Cripps Pink. This divergence suggests that physiological changes in aphids feeding on different cultivars, whether nutritional or defensive, and cultivar-specific volatile blends [64,65,66] underlie the suitability of host plants for different parasitoid species. Similar effects of host plant-mediated bottom-up influences on parasitoids have been documented in apple orchards and other fruit tree systems, highlighting key roles of cultivar resistance and aphid feeding behavior on parasitoid development and efficacy, influencing parameters such as sex ratio, fecundity, and survival [8,67].

Differences in aphid fresh mass, nutrient content, and defensive capabilities likely reflect plant-related variation in phloem sap composition, secondary metabolite profiles, and morphological traits [68,69,70,71]. Plant-mediated trade-offs, where distinct parasitoid species optimize performance on different cultivars, are consistent with findings from other systems that show strong host-plant-driven asymmetries in the success of biological control agents [34,72,73,74]. These results reinforce the concept that cultivar identity can cascade upward to influence parasitoid development, emergence rates, and reproductive investment, thereby shaping long-term population dynamics and field-level control efficacy [75,76,77].

The influence of seedling origin on parasitoid fitness extended beyond initial parasitism success, significantly shaping emergence rates and sex ratios. Notably, *A. ervi* emergence was markedly reduced on Elstar seedlings, while *A. matricariae* exhibited lower emergence on Granny Smith seedlings. Sex ratios also varied distinctly between the associated seedling origins. The occurrence of male-biased ratios was particularly evident for *A. ervi* on Granny Smith, Cox, and Kanzi seedlings, and for *A. matricariae* on Braeburn and Cox seedlings. These effects on fitness parameters beyond initial parasitism success further emphasize the differential bottom-up control exerted by seedling origin, which could influence long-term parasitoid population dynamics and biological control efficacy [78]. The observed seedling-origin-specific variations in parasitoid performance likely arise from differences in aphid nutritional quality and secondary metabolite profiles, which are known to influence parasitoid development, reproductive strategies, and host exploitation efficiency [72,74]. These findings are consistent with previous studies showing that apple cultivars can differentially influence aphid fitness and subsequently affect parasitoid development, reproductive success, and host preference [3,10]. To enhance IPM frameworks, future studies should focus on identifying the specific phytochemical or morphological traits underlying the observed interactions between apple seedling origins, aphids, and their parasitoids. Key areas of investigation include seedling-origin-specific volatile emissions, which are known to influence parasitoid foraging behavior and host selection [79,80,81]. Understanding these mechanisms may help develop targeted strategies for cultivar selection and orchard management, optimizing biological control of *D. plantaginea*.

Beyond bottom-up effects, this study highlights the contrasting outcomes of interspecific parasitoid interactions, ranging from synergistic to antagonistic. The co-release of *A. ervi* and *A. matricariae* enhanced parasitism rates and mummy counts across most seedling origins compared to solitary releases, notably on Braeburn and Golden Delicious, suggesting synergistic interactions driven by niche complementarity [82,83]. Differences in host-stage preferences, *A. matricariae* targeting early instars and *A. ervi* preferring older instars [21,22,24,84], facilitate complementary resource use and reduce direct interference, thereby enhancing aphid suppression at the orchard scale. Such complementarity is consistent with ecological theory predicting that multi-species assemblages with distinct niches can provide stronger and more stable regulation of herbivore populations [85,86,87]. Comparable synergistic effects have been recently confirmed in field release trials in apple orchards, where parasitoid co-deployments increased efficacy against *D. plantaginea* [88,89]. Host quality was reported to significantly influence these parasitoid preferences, as smaller aphids are less likely to escape parasitization, while larger aphids offer greater potential for parasitoid growth [90].

Conversely, on Cox seedlings, co-releases reduced parasitoid success relative to single-species treatments, indicating antagonistic outcomes likely due to interference competition or resource limitation under overlapping host exploitation [91,92]. These findings emphasize that while mixed parasitoid releases often strengthen pest regulation, under certain host–plant contexts they may diminish control efficiency. This is in line with theoretical and empirical evidence demonstrating that mixed parasitoid releases do not always yield superior outcomes and can sometimes lead to reduced control efficacy due to interspecific competition [33,93]. This observation has direct implications for IPM: parasitoid release strategies should not be universal but cultivar-specific, weighing the probabilities of synergistic benefits against potential antagonistic risks. Developing predictive models that integrate bottom-up host plant factors with top-down parasitoid behaviors could enhance release efficacy and cost-effectiveness [94].

Parasitoid host-selection is driven by a range of chemical and physical features of the plant on which the aphid is feeding [95,96,97]. In our study, parasitoid females revealed species-specific seedling-origin preferences within a multi-host plant system. The females of *A. ervi* exhibited a strong preference for aphids infesting Braeburn, Red Delicious, and Gala seedlings, whereas *A. matricariae* favored Cripps Pink seedling aphids. These contrasting choices likely reflect integrated responses to seedling-origin cues, such as host suitability, aphid nutritional quality, defensive traits, and volatile profiles, that shape parasitoid foraging strategies [34,98]. Such preferences are consistent with findings that plant chemical signals and aphid-host interactions differentially shape parasitoid host-selection behavior, as observed in other tritrophic systems [7,99,100]. These results highlight the critical role of host plant traits in optimizing parasitoid-based biocontrol strategies.

The strength of parasitoid-mediated indirect interactions in agroecosystems is closely tied to the suitability of plant-aphid associations. The results of this study provide a comprehensive understanding of parasitoid-mediated indirect interactions or “apparent competition”, among various aphid-infested apple seedlings, revealing how intraspecific plant variability influences tritrophic interaction dynamics. The observed asymmetrical interactions, where aphids associating with certain seedling origins like Gala and Braeburn act as predominant sources of parasitoids attacking aphids on adjacent host plants like Cox seedlings, align with broader ecological patterns of apparent competition in agroecosystems [16,42,43,101]. Such patterns may derive from host plant-specific differences in structure, volatile and nutrition profiles influencing parasitoid attraction and retention, as highlighted by recent studies on plant-mediated insect interactions [102,103].

For instance, parasitoids developing on Gala aphids appeared more likely to attack aphids on Cox than vice versa. Such asymmetrical interactions are common in apparent competition scenarios and are consistent with metapopulation theory, which explains how moving between different habitats and the presence of source populations can shape overall population patterns [104,105]. The varying levels of ‘self-sourcing’ of parasitoids suggest differences in parasitoid movement and retention among host plants, with Gala and Braeburn seedlings showing high retention and Red Delicious showing high exchange. The fact that aphid populations on certain seedling origins act as sources of parasitoids, which then disperse to others, suggests that manipulating plant diversity at the orchard level could be a valuable strategy for enhancing biological control across the entire orchard [106]. The significance of habitat composition and plant diversity in promoting parasitoid diversity and efficacy has been increasingly recognized as a cornerstone of effective biological control in orchard ecosystems [107,108].

While controlled laboratory experiments provide critical insights under manageable settings, the complexity of field environments necessitates cautious extrapolation. Abiotic factors such as temperature fluctuations and humidity, along with biotic interactions like hyperparasitism, ant attendance, and multi-predator effects, modulate aphid-parasitoid dynamics in situ [109,110,111]. Spatial scale effects influencing parasitoid dispersal and persistence require further elucidation through landscape-scale studies and long-term monitoring [112,113,114]. Therefore, long-term trials incorporating orchard-scale replication, environmental variability, and ecological interactions beyond parasitoids (e.g., coccinellids, syrphids, spiders) are essential for translating laboratory findings into practical applications [115,116].

## 5. Conclusions

Our findings demonstrate that both bottom-up plant effects and top-down parasitoid interactions should be considered together to optimize *D. plantaginea* control. These interactions are neither uniform nor predictable from parasitoid performance alone but arise from the dynamic interplay of host plant identity, host aphid quality, and parasitoid behavior. Adapting parasitoid deployment strategies to cultivar-specific contexts, while leveraging synergistic interactions and minimizing antagonism, holds promise for strengthening IPM interventions. Such integrative ecological approaches may lead to more sustainable and effective pest management practices [117]. Future work that links cultivar-driven plant–insect traits with parasitoid network dynamics under realistic field conditions will be essential for moving toward sustainable, knowledge-intensive management of rosy apple aphid populations.

## Figures and Tables

**Table 1:** Number of mummies (mean ± SE) of *Aphidius ervi* and *Aphidius matricariae* on *Dysaphis plantaginea* infesting different apple cultivars in mono-cultivar system. Different letters indicate significant differences between aphid-infested apple cultivars (GLM and Tukey test, *p* ≤ 0.05).

Cultivars	*A. ervi*	*A. matricariae*
Braeburn	13.7 ± 2.0 ab	10.3 ± 0.8 ab
Cox	13.2 ± 1.3 ab	7.3 ± 0.9 bcd
Cripps Pink	9.0 ± 0.7 bc	17.9 ± 3.0 a
Elstar	7.8 ± 1.6 c	7.8 ± 1.4 bcd
Gala	6.4 ± 0.6 c	4.3 ± 0.8 d
Golden Delicious	24.4 ± 4.0 a	4.1 ± 0.9 d
Granny Smith	7.5 ± 1.0 bc	5.2 ± 0.7 cd
Kanzi	5.2 ± 0.7 c	5.2 ± 0.8 cd
Red Delicious	18.2 ± 1.4 a	7.6 ± 0.9 bcd
Topaz	13.0 ± 1.1 ab	8.1 ± 0.7 bc
F_9,90_	11.32	9.80
*p*	<0.001	<0.001

**Table 2:** Parasitism rate (%, mean ± SE) of *Aphidius ervi* and *Aphidius matricariae* on rosy apple aphids across different apple cultivars. Different letters indicate significant differences in the parasitism rates between associated cultivars (Kruskal–Wallis and Dunn’s tests, *p* ≤ 0.05).

Cultivars	*A. ervi*	*A. matricariae*
Braeburn	23.0 ± 4.1 ab	9.7 ± 0.8 bc
Cox	14.2 ± 2.1 bc	14.3 ± 2.6 ab
Cripps Pink	6.4 ± 0.5 d	24.3 ± 3.4 a
Elstar	10.4 ± 2.9 cd	11.4 ± 2.7 bc
Gala	8.0 ± 0.8 cd	4.7 ± 1.1 e
Golden Delicious	38.7 ± 5.8 a	6.1 ± 1.3 de
Granny Smith	7.8 ± 1.2 d	6.4 ± 0.8 cde
Kanzi	6.4 ± 1.0 d	5.3 ± 0.7 de
Red Delicious	31.0 ± 3.8 a	11.2 ± 1.4 b
Topaz	14.6 ± 1.6 bc	8.1 ± 0.8 bcd

## Data Availability

The original contributions presented in this study are included in the article. Further inquiries can be directed to the corresponding author.
